# Supplementary material for: Extrinsic nature of the broad photoluminescence in lead iodide-based Ruddlesden–Popper perovskites
Source: Nat Commun. 2020 May 11;11:2344. doi: 10.1038/s41467-020-15970-x (PMC7214453; doi:10.1038/s41467-020-15970-x)
Supplement: Supplementary file 1 — Supplementary Information [file 41467_2020_15970_MOESM1_ESM.pdf]

Supplementary Information for: Extrinsic nature of the broad photoluminescence in lead iodide-based Ruddlesden Popper perovskites

Simon Kahmann,[†] Eelco K. Tekelenburg,[†] Herman Duim,[†] Machteld E. Kamminga[†,‡] and Maria A. Loi[†,*]

[†] *Zernike Institute for Advanced Materials, University of Groningen, Nijenborgh 4, 9747 AG, Groningen, The Netherlands*

[‡] *Current address: Department of Chemistry, Inorganic Chemistry Laboratory, University of Oxford, South Parks Road, Oxford OX1 3QR, United Kingdom*

E-mail: m.a.loi@rug.nl

Supplementary Figures

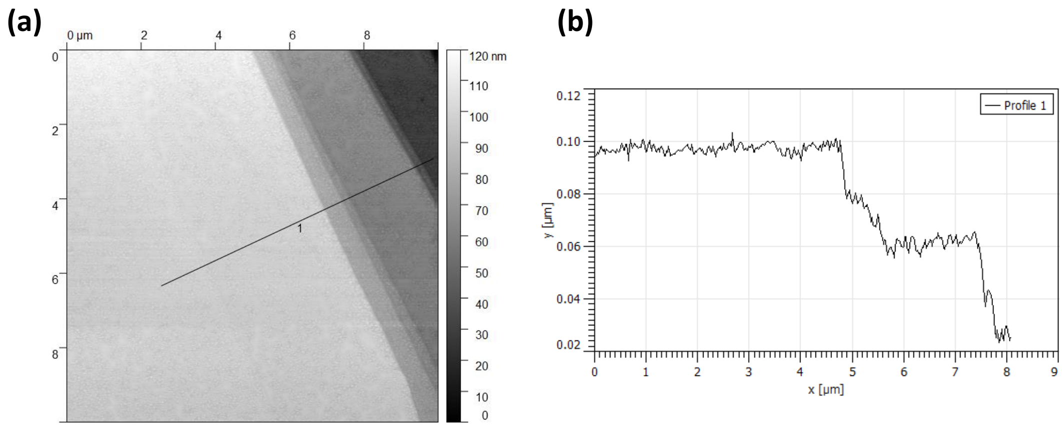

Supplementary Figure 1: AFM micrograph near the edge (see top tight corner) of a crystal flake including a line scan as indicated in black (a). The extracted height profile is shown in (b). The layered nature of the material can be inferred from formed terraces towards the edge of the flakes.

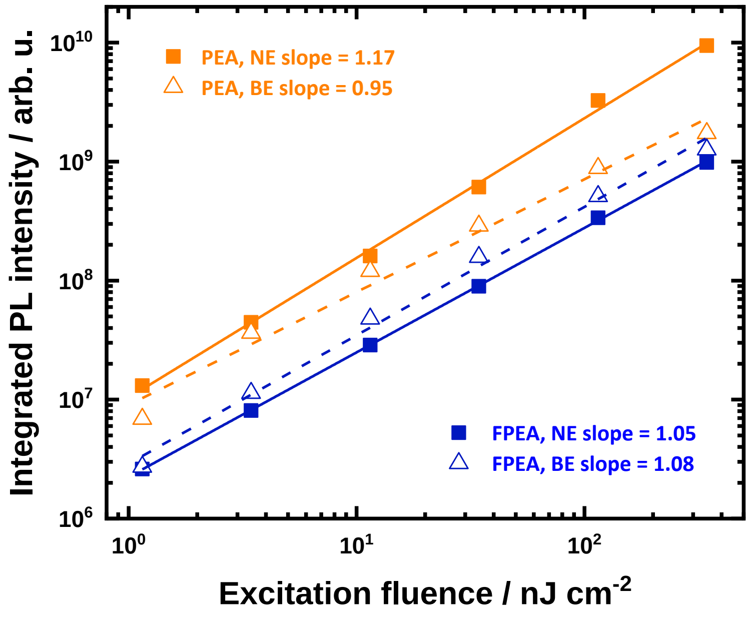

Supplementary Figure 2: Fluence dependent PL intensity of the NE and BE for both types of crystals. Over a broad range of intensities, the PL scales linearly with the excitation fluence indicating first order processes.

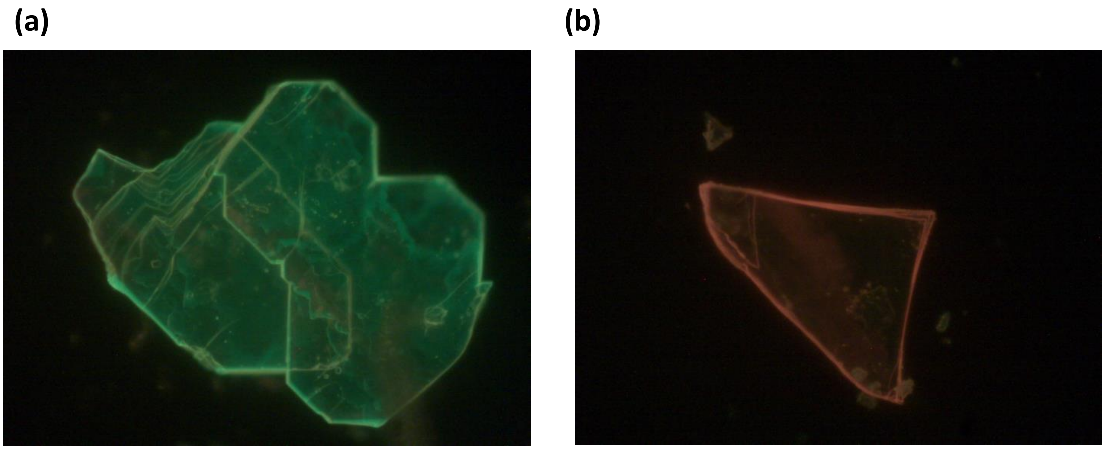

Supplementary Figure 3: Wide-field PL micrographs ($230 \times 175\,\mu$m) show how some flakes (both FPEA) appear bright green over their entire area (a), whilst other flakes exhibit a distinctly red-shifted emission (b).

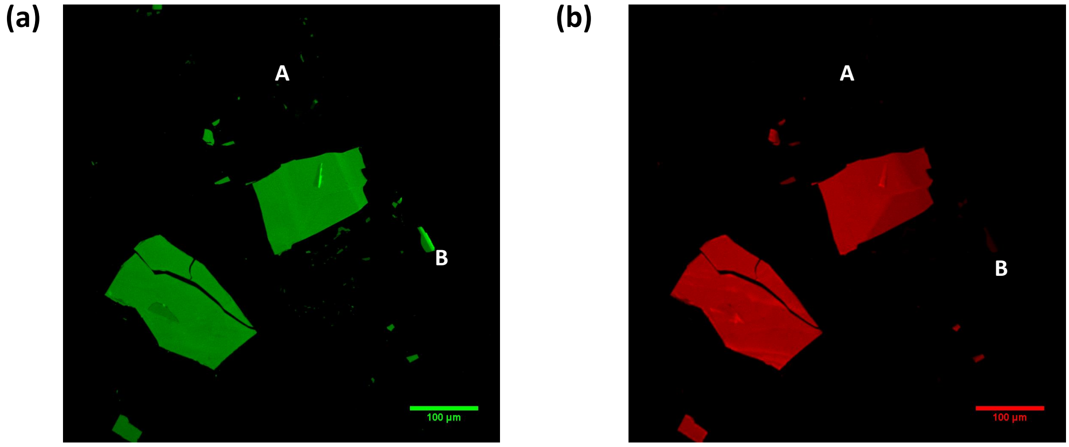

Supplementary Figure 4: Confocal PL micrographs of PEA flakes measured through the green (a) and red (b) channel. As indicated by the letters, bright green areas, which are very prominent for the debris, correspond to areas of hardly detectable red emission. Conversely, the large flakes in the centre and bottom left corner exhibit bright red luminescence and reduced contributions (relative to the small flakes) in the green channel.

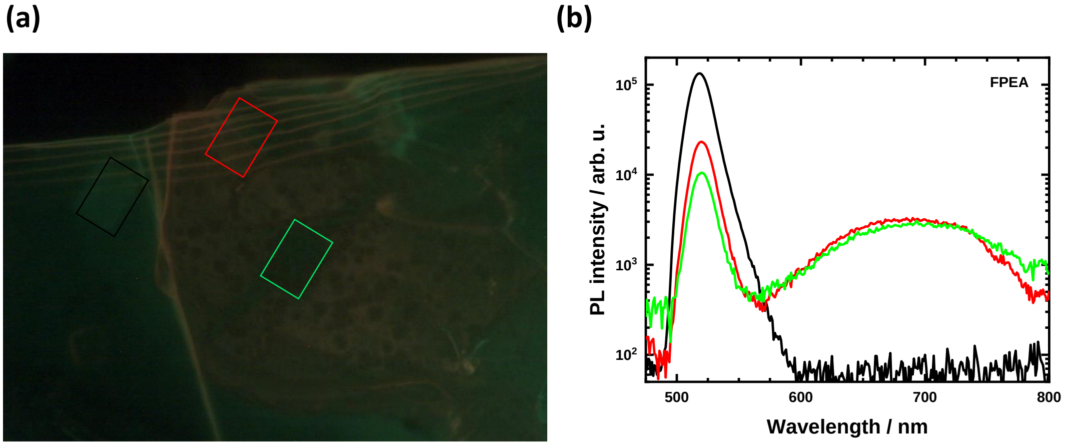

Supplementary Figure 5: Wide-field photoluminescence micrograph (230×175 μm) of an FPEA flake (a). The coloured rectangles indicate approximate areas, where the PL spectra depicted in (b) were taken. These representative cases highlight the absence of any correlation between the edges of crystal flakes and the broad emission band.

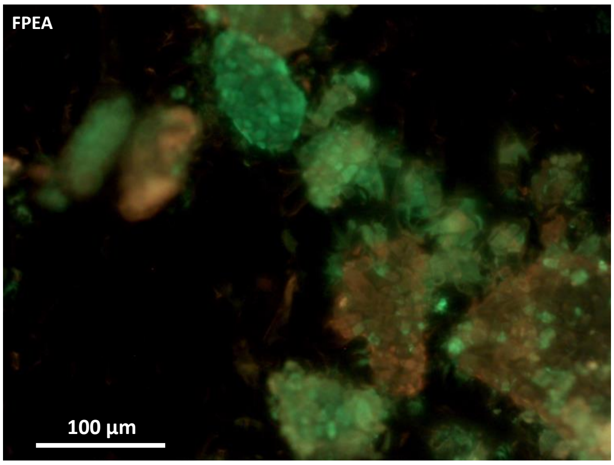

Supplementary Figure 6: Wide-field photoluminescence micrograph of crystal flakes that were crushed between two quartz plates. The emission of some crushed flakes remains bright green (*e.g.* centre top), whilst others' luminescence remains red-shifted (centre right).

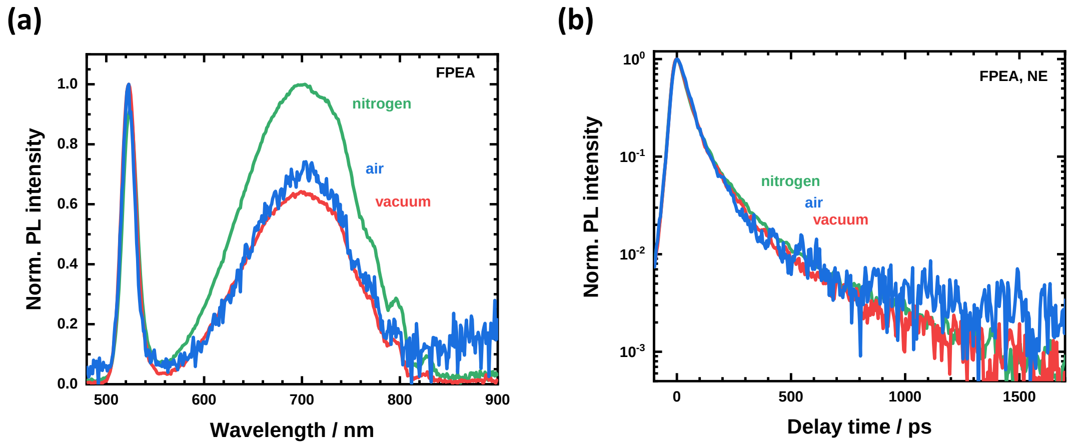

Supplementary Figure 7: Normalised steady state spectra (CLSM, uncalibrated) of FPEA crystals exposed to different atmospheres. The spectra in (a) exhibit small variations due to non-ideal re-alignment between the different experiments (the same holds for the variations in signal to noise ratio). Importantly, though, the NE decays with the identical lifetime, indicating the lack of impact of different atmospheres.

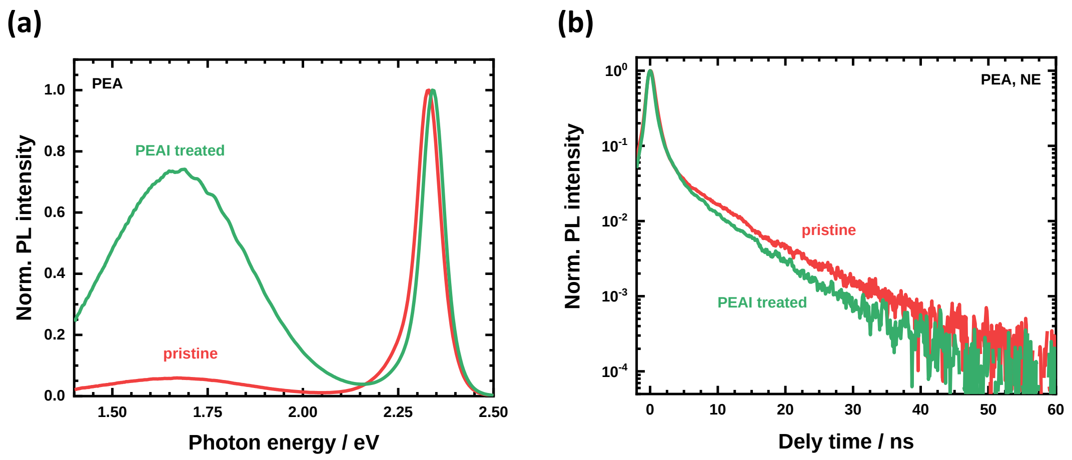

Supplementary Figure 8: Steady state (a) and transient (b) PL spectra of PEA flakes prior and after exposure to PEAI dissolved in IPA. The BE cannot be suppressed by further exposure to the organic molecules.

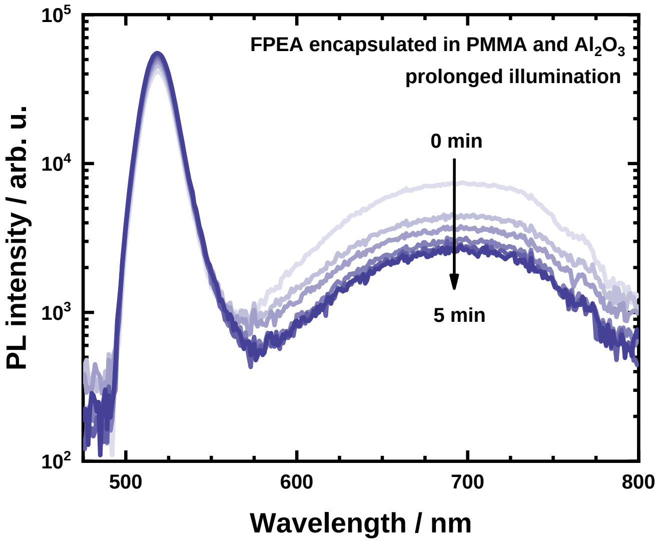

Supplementary Figure 9: Steady state PL of an encapsulated FPEA flake (uncalibrated spectra taken with the CLSM). The BE is clearly visible from the start and prolonged illumination reduces its intensity, as observed without encapsulation.

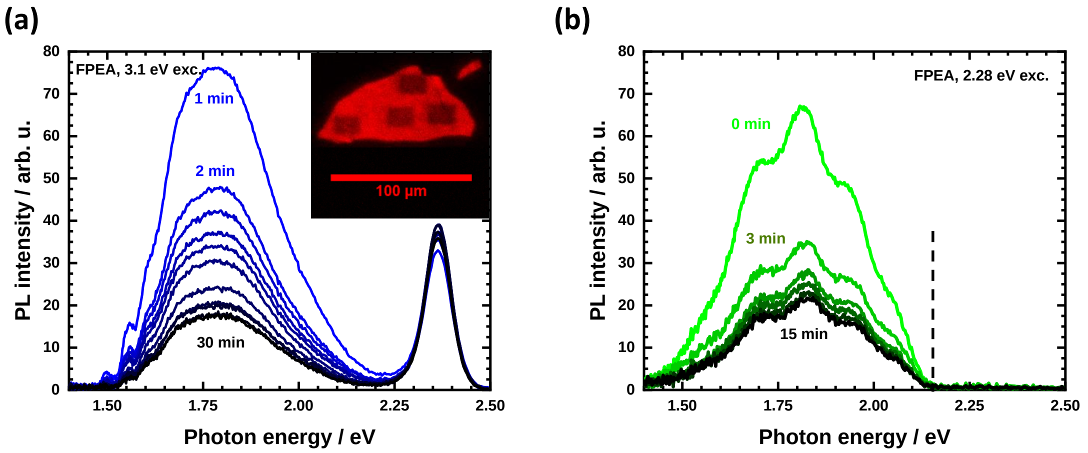

Supplementary Figure 10: Prolonged illumination of the crystals reduces the BE intensity in an irreversible process (a) (uncalibrated spectra). The inset shows a red-channel micrograph including dark patches of previously illuminated areas. Prolonged laser exposure also reduces the BE intensity when nominally below-gap photons are used (b) (the dashed line indicates the long pass filter used to cut-off the excitation. The filter is also responsible for the intensity modulation.).

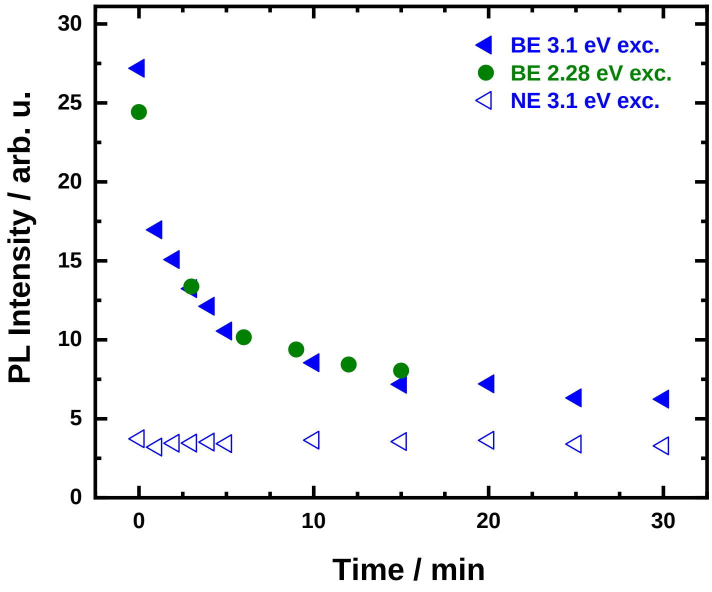

Supplementary Figure 11: Tracking the peak intensity of Figure S10 over time reveals the same BE reduction independent of the incident photon energy. Importantly, the NE does not increase simultaneously, but remains relatively stable. BE healing does thus not suppress the non-radiative decay of free excitons.

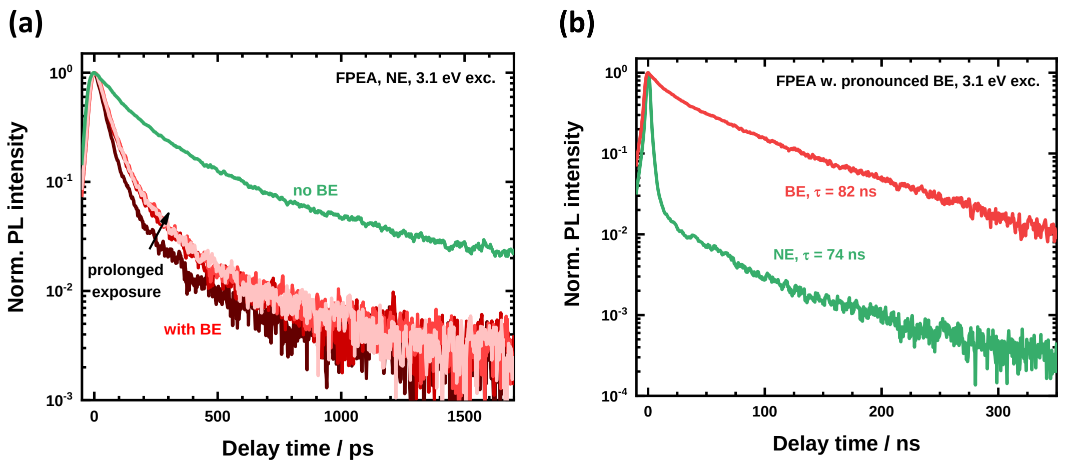

Supplementary Figure 12: Time resolved PL of FPEA at room temperature. The PL of the NE decays much faster in areas of pronounced BE (a), but prolonged illumination only has a small impact on its early decay dynamics. The BE exhibits a significantly longer lifetime that the initial NE decay, but as shown in (b), there is also a weak tail at long delays observable for the NE.

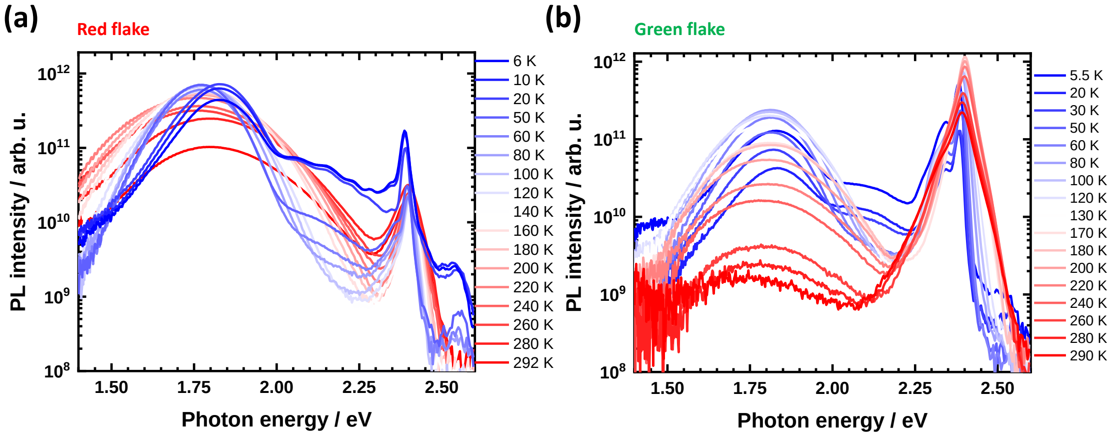

Supplementary Figure 13: Steady state PL spectra of a red flake (a) and a green flake (b) upon temperature variation. Data replotted from Figure 4 of the main text.

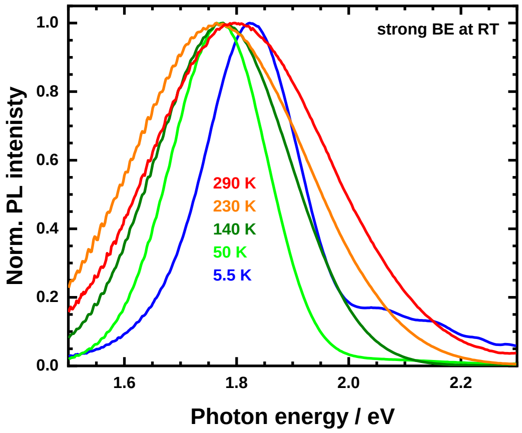

Supplementary Figure 14: Normalised steady state PL spectra of an area with pronounced BE at room temperature for selected temperatures. The BE peaking at 1.82 eV at 5 K not only broadens upon temperature increase, but can also not be described by a single Gaussian peak any more.

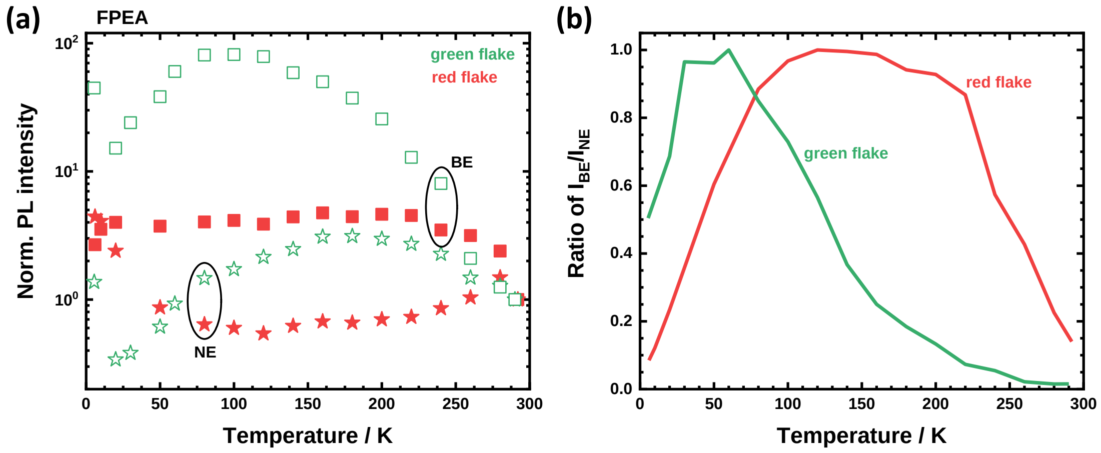

Supplementary Figure 15: Intensity of the BE (squares) and NE (stars) normalised to their room temperature value for a green and a red FPEA flake on a semi-logarithmic scale (a). Initial temperature decrease brightens all signals, but below 275 K different trends emerge for the flakes and emission bands. The ratio of BE:NE is shown in (b). Both cases exhibit a maximum, which lies around 50 K for the green and 150 K for the red flake.

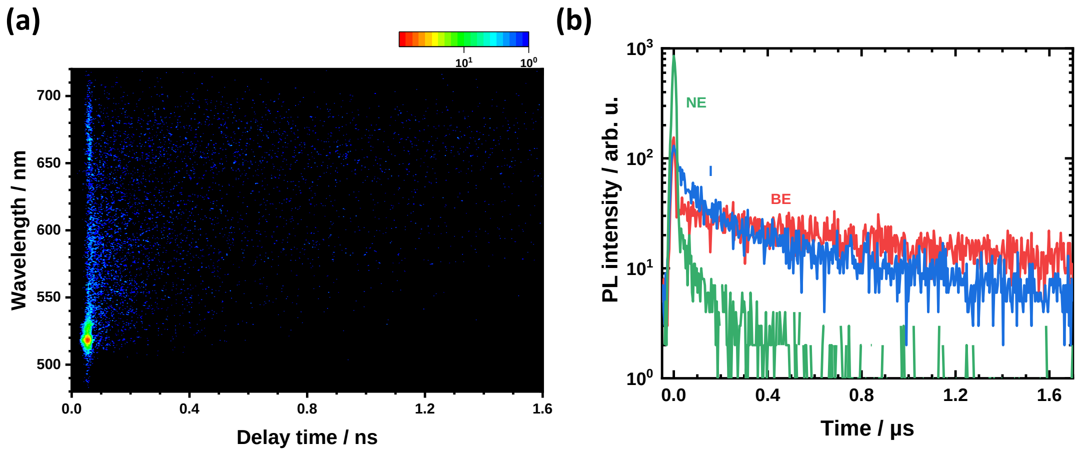

Supplementary Figure 16: Streak camera image of an FPEA red flake at 20 K (a) with the extracted transients of the emission bands shown in (b). The three band exhibit distinctly different decays, underlining their origin to be distinct states.

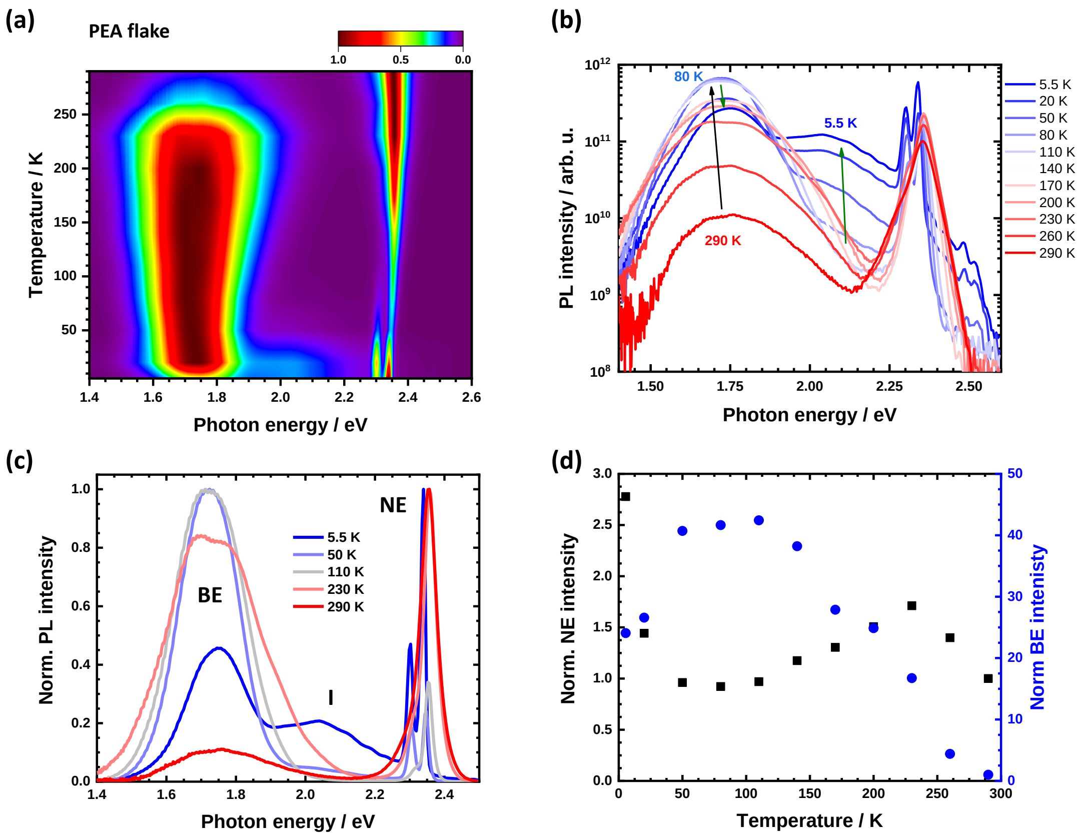

Supplementary Figure 17: Temperature dependent PL spectra of a PEA flake with moderate BE at room temperature. The normalised false colour plot (a) exhibits the previously discussed brightening of the BE upon temperature reduction, the NE peak splitting and the strong emergence of luminescence from I. The absolute intensity data in (b) highlights the strong increase of the BE down to approximately 80 K, below which I becomes increasingly dominant and the BE intensity slightly diminishes. Selected spectra from (a) are plotted in (c) to underline the changing sub-structure of both the BE and the NE. Neither can be explained by assuming a single emitting state. The extracted intensities of the NE and BE are given in (d), showing the expected strong resemblance to the green flake of FPEA.

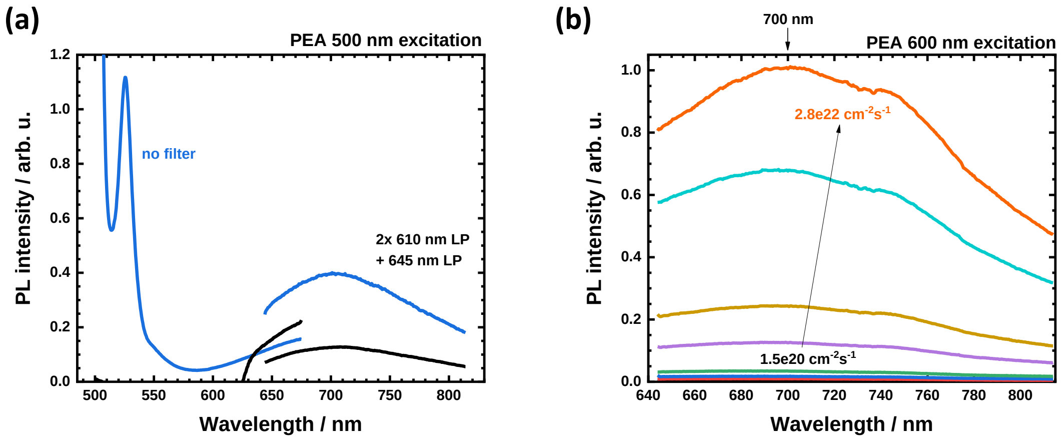

Supplementary Figure 18: Excitation of PEA flakes at 500 (a) and 600 nm (b). Two measurements are needed to cover the spectral range of the NE and BE. Long pass filters were used to suppress scattered laser light, but simultaneously denying the detection of the NE (a). The BE upon 600 nm excitation was detected over two orders of magnitude in photon fluence (b).

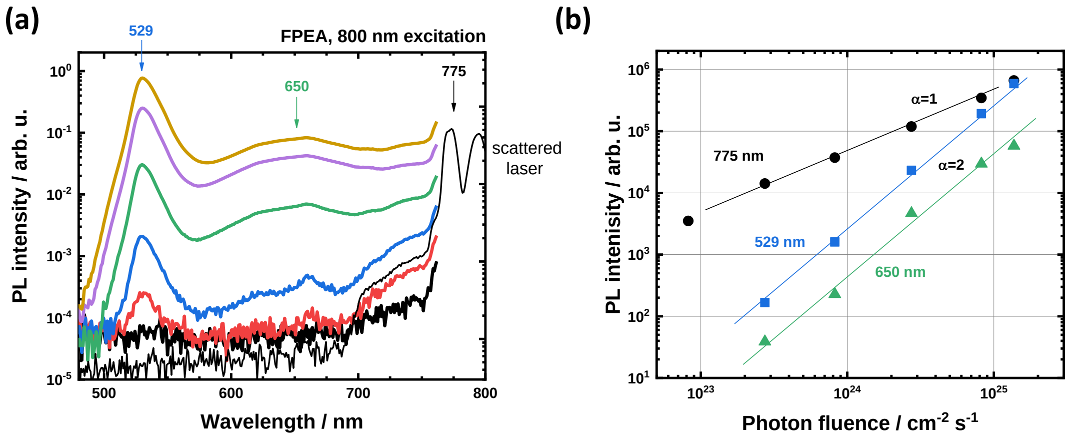

Supplementary Figure 19: PL spectra of an FPEA flake upon 800 nm excitation (a). The extracted PL intensity at three selected points in (b) displays the quadratic fluence dependence of both the NE and BE, which contrast with the linearly scaling intensity of scattered laser light at 775 nm.

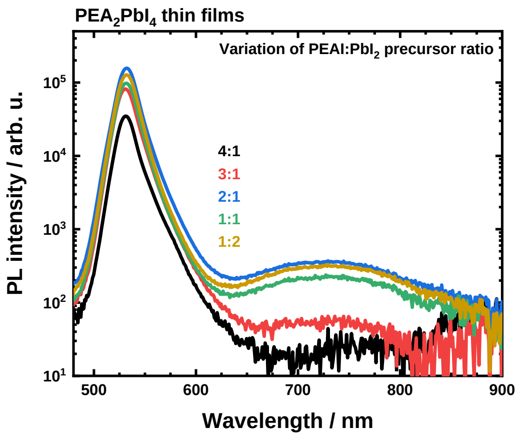

Supplementary Figure 20: Photoluminescence spectra of thin films of PEA_2PbI_4 cast from solutions of different PEAI:PbI_2 precursor ratio, as previously discussed by our group.[1] A depletion of PEAI suppresses the formation of the broad emission band.

Supplementary Note 1

Power dependent PL spectra allow for deducing the reaction order of the radiative recombination. Supplementary Figure 2 displays the respective data for both types of crystals measured for both the NE and BE. In each case, the PL scales approximately linearly with incident laser power, indicating first order recombination. This allows, for example, for excluding free carrier recombination as the origin of the luminescence. The responsible processes are thus either due to excitons or trap-mediated recombination. The latter is often associated with a linear behaviour that saturates under high fluence. However, whilst the BE of PEA seems to run into saturation, it cannot be observed in the case of FPEA. The absence of saturation has led some authors to exclude trap-mediated recombination as the origin of the BE.

Supplementary Note 2

Wide-field PL micrographs of the cleaved crystals show a broad variety of colours, depending on the considered flakes. Two examples or FPEA are given in Supplementary Figure 3, for which the flake in (a) offers bright green luminescence (green flake) and the one in (b) appears much fainter and red-shifted (red flake). In both cases the edges appear brighter due to waveguiding effects, but retain the same colour.

Local probing of the PL spectra with a spot diameter of one square micrometre allows for determining whether the perceived colour of the flakes is governed by effects on the edges. As indicated by the steady state spectra in Supplementary Figure 5, the BE can

be as dominant on the edge (red) as in the centre of the crystal (green), but may even be absent on the edge (black), excluding previously invoked edge states as the origin of the BE.

A simple way to increase the surface of crystal flakes and to thereby monitor whether potential surface defects are dominant in the formation of the red-shifted BE, is to physically crush the flakes. The wide-field PL micrograph in Supplementary Figure 6, however, clearly shows that the bright green or red-shifted character of the crystals is retained by their debris. Some crushed material shows distinct red-shifted emission, but other areas remain bight green.

Several reports showed that the PL characteristics of perovskites can be significantly affected by the atmosphere of their surroundings. To monitor a possible suppression of surface defects and its effect on the BE we exposed the cleaved crystals to different environments (Supplementary Figure 7). Since the BE is clearly visible in all cases and the transients of the NE remain unaffected, this serves as additional evidence to exclude surface processes.

Surface treatment of organic ligands *e.g.* PEA^+ on PEA-based 2D perovskites was previously shown to increase the luminescence yield and boost device performance through passivation.[1,2] Treatment of PEA flakes with 10 mmol of PEAI dissolved in isoprpyl alcohol does, however, not suppress the BE. In contrast, we find a much stronger BE in steady state (Supplementary Figure 8 (a)) and a slightly reduced lifetime of the NE (b). This could be due to a large point-to-point variation and non-ideal re-alignment, but as discussed for thin films, excess PEA^+ can also increase the BE intensity.[1]

Encapsulation of the flakes can suppress laser damage on the surface.[3] However, when

the flakes are encapsulated under a thin film of PMMA and ALD-processed aluminium oxide, there is no effect on the BE beyond the light-induced healing discussed below.

Supplementary Note 3

Supplementary Figure 10 (a) displays the obtained spectra of a red flake upon continuous exposure to 3.1 eV light in the CLSM set-up. The intensity of the BE decreases over the first ten minutes and saturates afterwards. Simultaneously, the NE shows some minor variation, but by far not as pronounced as the BE. Importantly, the NE and BE behaviour do not correlate (as shown in Supplementary Figure 11). The same trend in BE intensity is observed for continuous excitation at 2.28 eV, i.e. below the absorption onset of the material (b). Given that these measurements were conducted in CLSM geometry under a tightly focused laser spot, we cannot exclude the latter to lead to two-photon absorption. Notably, as indicated by the inset of Supplementary Figure 10 (a), the red-channel PL of previously exposed areas (darker rectangles) remains lower than initially. The change in BE intensity is thus irreversible (on the timescale of the experiment), but BE suppression does not strongly affect the NE signal. Such variation underlines the BE to be of extrinsic origin.

Time-resolved photoluminescence reveals further striking differences between regions dominated by the BE and those exhibiting mostly NE. Supplementary Figure 12 (a) shows the transients of the latter on a picosecond timescale. In case of predominantly narrow emission, the PL decays much slower than in regions of pronounced BE. Conse-

quently, red regions exhibit additional channels for non-radiative decay of free excitons. Prolonged laser exposure only slightly increases the NE lifetime, which is in accordance with the negligible effect on the steady state intensity given in Supplementary Figure 11. In other words, although the red regions coincide with additional decay channels for free excitons, the suppression of the BE is not identical with a passivation of these channels. The lifetime of the BE lies on a longer timescale of hundreds of nanoseconds, as shown in Supplementary Figure 12 (b). In the example of FPEA, the average lifetime was determined to 82 ns. Strikingly, the NE measured in the same spot exhibits a weak, but distinct tail at long delay with a lifetime of 74 ns.

Supplementary Note 4

The complete data used for Figure 4 of the main text is given in Supplementary Figure 13. Towards room temperature, the BE vanishes for the green flake, but for the red flakes the broad emission cannot be explained by assuming solely one peak (Supplementary Figur. 14). At 5 K the BE consists of only one emissive state centred around 1.82 eV (along with I at 2.15 eV). Towards room temperature, however, the BE loses the symmetry and becomes more stretched on its high energy side.

The integrated intensity of the two main emission bands are plotted in Supplementary Figure 14 (a) for a red and a green flake. Whilst all signals brighten upon initial temperature reduction, the trends differ significantly below 275 K. In the case of a green flake, the BE intensity increases strongly and continuously down to approximately 80 K,

below which the emission is reduced likely by the increasing presence of I, as discussed in the main text. The green flakes NE similar brightens over a certain range, but its intensity already diminishes below approximately 160 K. In contrast, the red flakes emission bands exhibit a comparatively small dependence on the temperature down to 80 K. Whilst the BE brightens from RT down to 220 K it then remains virtually constant over the entire range with a small reduction below 80 K. The NE intensity slightly decrease already from 275 K, but starts to increase again below 80 K. Whilst the two different kinds of flakes thus behave differently, there seems to be a general impact on the NE and BE by the emergence of I. Plotting the BE to NE intensity ratio is often used to deduce trapping and detrapping energy barriers, especially in the framework of STEs.[4] We show the respective data in Supplementary Figure 14 (b) in a double-linear plot. The stark contrast described above manifests in two strikingly different trends, again indicating that the observed emission cannot be of an intrinsic origin.

As noted above for low temperature spectra (*e.g.* Supplementary Figure 13 & 14), the region around 2.15 eV shows a distinct emission band forming below approximately 80 K. Luminescence in this region is neither due to the NE nor BE. This intermediate state I is pronounced for both red flakes and green flakes and similarly forms in PEA (see below). Accordingly, transient measurements reveal decay dynamics of I that are distinct from those of the NE and BE. Supplementary Figure 16 displays the streak camera image from a red flake taken at 20 K (a) along the extracted transients for the three states in (b). The large delay shows how most of the NE intensity decays quickly, whilst both the BE and I show more persisting signals on the μs range.

Supplementary Note 5

The direct excitation of the BE with photons of energy below the band gap occurred on a second set-up, as discussed in the experimental section. The finer grating of this set-up does not allow for observing both the NE and BE region in a single measurement and we thus show the entire spectral region with two separate measurements in Supplementary Figure 18. The blue lines in (a) indicate the emission of a PEA flake upon 500 nm excitation (above gap) without the use of filters in the collection path (see experimental). The NE and BE are both visible, but the laser light is clearly much stronger. In order to avoid erroneous results, two 610 nm and one 645 nm long pass filters were inserted in the collection path to suppress the detection of scattered laser light (black curves). Fluence dependent excitation at 600 nm was then successfully used to directly excite the BE in either type of crystal with, for which the obtianed curves of the PEA flakes are shown in Supplementary Figure 18 (b). The intensity used in Figure 5 of the main text was extracted at 700 nm, as indicated.

The direct excitation of the BE with photons below the band gap can be distinguished from the case of two-photon absorption by their dependence on the excitation fluence, as discussed in the main text. Given the high fluences needed in this experiment, the high quality 775 nm short pass filter does not completely suppress the detection of scattered laser light, as shown by the thin black line in Supplementary Figure 19 (a). The obtained spectra of the flakes contain both the sample PL and this scattered light. Nonetheless, the PL intensity of the NE and BE both clearly scales with a power of 2, as expected from a two-photon process, whilst the laser scales linearly (see Supplementary Figure 19)

Analogous results were obtained when measuring flakes of either PEA or FPEA independent of the sample being in air or vacuum as well as the degree of BE it exhibits.

Supplementary References

[1] Duim, H., Adjokatse, S., Kahmann, S., ten Brink, G. H. & Loi, M. A. The Impact of Stoichiometry on the Photophysical Properties of RuddlesdenPopper Perovskites. *Adv. Funct. Mater.* **30**, 1907505 (2020).

[2] Duim, H. *et al.* Mechanism of surface passivation of methylammonium lead tribromide single crystals by benzylamine. *Appl. Phys. Rev.* **6**, 031401 (2019).

[3] Fang, H.-H. *et al.* Unravelling Light-Induced Degradation of Layered Perovskite Crystals and Design of Efficient Encapsulation for Improved Photostability. *Adv. Funct. Mater.* **28**, 1800305 (2018).

[4] Itoh, N. & Stoneham, M. *Materials Modification by Electronic Excitation* (Cambridge University Press, Cambridge, 2000).
